# An inducible model for genetic manipulation and fate-tracing of PDGFRβ-expressing fibrogenic cells in the liver

**DOI:** 10.1038/s41598-023-34353-y

**Published:** 2023-05-05

**Authors:** Florian Hamberger, Young-Seon Mederacke, Ingmar Mederacke

**Affiliations:** Department of Gastroenterology, Hepatology, Infectious Diseases and Endocrinology, Hannover Medical School, Carl-Neuberg-Str. 1, 30625 Hannover, Germany

**Keywords:** Cancer, Cell biology, Developmental biology, Molecular biology, Gastroenterology, Medical research

## Abstract

Myofibroblasts are the source of extracellular matrix protein during liver fibrogenesis. Fibroblasts, hepatic stellate cells (HSCs) and vascular smooth muscle cells are mesenchymal subpopulations in the liver that are characterized by the expression of PDGFRβ and contribute to the pool of these myofibroblasts. Conditional knockout models are important to better understand the function of specific liver cell populations including mesenchymal cells. While there is a limited number of constitutive mouse models for liver mesenchymal cell specific transgene expression, there is no established model for inducible gene targeting in HSCs or PDGFRβ-expressing mesenchymal cell populations in the liver. To address this, we investigated whether the tamoxifen inducible PDGFRβ-P2A-CreER^T2^ mouse can be used as a reliable tool to specifically express transgens in liver mesenchymal cells. Our data demonstrate, that PDGFRβ-P2A-CreER^T2^ specifically and efficiently marks over 90% of retinoid positive HSCs in healthy and fibrotic liver in mice upon tamoxifen injection, and that those cells give rise to *Col1a1*-expressing myofibroblasts in different models of liver fibrosis. Together with a negligible background recombination of only about 0.33%, this confirms that the PDGFRβ-P2A-CreER^T2^ mouse is nearly as efficient as established constitutive LratCre and PDGFRβ-Cre mouse models for recombination in HSCs, and that it is a powerful model for mesenchymal liver cell studies that require an inducible Cre approach.

## Introduction

Liver fibrosis and its end stage liver cirrhosis are a major cause of patient mortality worldwide^1^. However, despite extensive research into the underlying mechanisms of fibrosis development, there are still no antifibrotic drugs to date^2^. Hepatic stellate cells (HSCs), a pericyte-like cell population in the liver, could be identified as a dominant source of myofibroblasts in the liver independent of liver damage etiology^3^. Accordingly, deletion of Col1a1 in LratCre-positive HSCs significantly reduced the deposition of fibrillar collagen in the liver^4^. Moreover, single-cell RNA sequencing study revealed that HSCs are not a functionally homogenous cell population, but show spatial zonation of HSCs with central-vein-associated HSCs being the dominant collagen-producing cells in contrast to portal vein-associated HSCs^5^, and that inhibition of central-vein-associated HSCs is a promising approach to therapy in non-alcoholic steatohepatitis (NASH). A recent study identified three different mesenchymal subpopulations—fibroblasts, HSCs and vascular smooth muscle cells (VSMC)—by using a platelet-derived growth factor receptor beta (Pdgfrb)-GFP knockin reporter mouse^5^. Research into the function and behavior of the different mesenchymal cell populations, though, is limited by the availability of animal and cell culture models. Constitutive Cre transgenic mice are available that can be used in combination with fluorescent Cre-reporter mice to study liver fibrogenic cell populations^6^. While the lecithin retinol acyltransferase (Lrat)-Cre transgenic mouse can be used to mark HSCs^3^, PDGFRβCre transgenic mice label all mesenchymal cells populations^6,7^. Constitutive expression can have downsides as certain gene modifications are embryonically lethal, or in the case that the timing of genetic modification is of interest^8^. In these cases, an inducible Cre would add a valuable research tool. One of the most commonly used systems for inducible Cre expression is the CreER system, which combines the Cre recombinase with a tamoxifen binding domain^9^. In this model the binding of tamoxifen, an artificial estrogen-receptor antagonist^10^, to the CreER fusion protein facilitates the translocation of it from the cytoplasm to the nucleus, where the Cre can exert its function. In this way the induction of Cre activity can be controlled by tamoxifen application.

As there is no established inducible mouse model for the expression of transgens in the different hepatic mesenchymal cell populations to date, we sought to investigate whether the recently published PDGFRβ-P2A-CreERT2 transgenic mouse that efficiently labels pericytes in the retina and brain^11^ also marks mesenchymal cell populations in the liver and therefore could constitute a viable model for inducible gene deletion.

## Results

### PDGFRβ-P2A-CreER^T2^ efficiently labels retinoid positive HSCs in the liver upon tamoxifen induction

**Figure 1:**
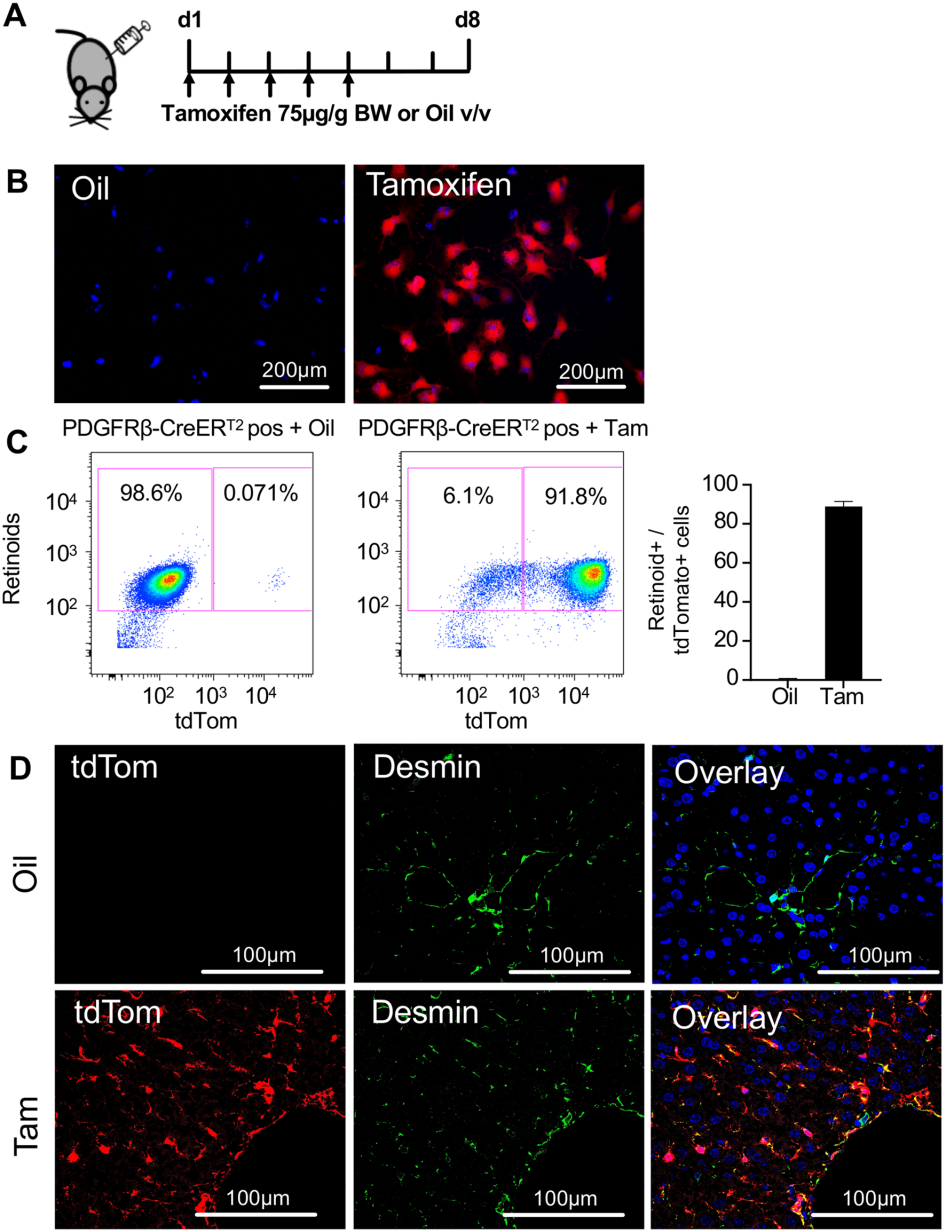
PDGFRβ-P2A-CreER^T2^ efficiently labels retinoid positive HSCs in the liver upon tamoxifen induced Cre activation. (**A**) PDGFRβ-P2A-CreER^T2^ mice were crossed with mice containing a tdTomato (tdTom) Cre reporter. Cre activity was induced by tamoxifen injection (i.p.) at 75 µg/g BW for five consecutive days. Animals were sacrificed three days after the last tamoxifen or oil injection, respectively. (**B**,**C**) Fluorescent images (**B**) and pseudocolor plots (**C**) of freshly isolated HSCs from PDGFRβ-P2A-CreER^T2^ x tdTomato mice that received tamoxifen or oil show Cre-mediated tdTomato expression only in tamoxifen treated animals (n = 4). (**D**) Fluorescent staining shows co-localization of desmin with PDGFRβ-P2A-CreER^T2^ induced tdTomato expression.

First, we were interested whether HSCs are efficiently labeled by PDGFRβ-P2A-CreER^T2^. Therefore, we crossbred the PDGFRβ-P2A-CreER^T2^ mouse line with the red fluorescent tdTomato Cre reporter mouse line to generate PDGFRβ-P2A-CreER^T2^ x tdTomato animals. These mice were injected with tamoxifen or oil for five consecutive days. Subsequently, the liver was harvested at day 8 for either immunofluorescent staining or isolation of HSCs by cell sorting (Fig. 1A). Analysis by fluorescent microscopy did not detect any expression of tdTomato Cre reporter in oil-injected control mice. In contrast, PDGFRβ-P2A-CreER^T2^ x tdTomato mice that received tamoxifen showed strong tdTomato expression in the majority of retinoid positive HSCs (Fig. 1B). To determine the percentage of HSCs that are labelled by PDGFRβ-P2A-CreER^T2^, we analyzed freshly isolated HSCs by flow cytometry. In tamoxifen injected animals, we observed that a mean of 88.85 ± 2.57% HSCs (violet A positive) were also positive for tdTomato indicating a high efficiency of PDGFRβ-P2A-CreER^T2^ for HSCs. In contrast, oil-injected PDGFRβ-P2A-CreER^T2^ x tdTomato animals only had a very low level of tdTomato labelled HSCs (0.33 ± 0.47%), indicating a neglectable background recombination (Fig. 1C, Supplemental Fig. 1A). Furthermore, immunofluorescent staining for desmin on frozen liver sections of PDGFRβ-P2A-CreER^T2^ x tdTomato mice showed a strong overlap of tdTomato fluorescence in untreated and fibrotic liver (Fig. 1D, Supplemental Fig. 1B–D). Importantly, PDGFRβ-P2A-CreER^T2^ induced tdTomato expression was detected by fluorescent microscopy up to one year after tamoxifen-induction in HSCs in the liver (Supplemental Fig. 1E).

### PDGFRβ-P2A-CreER^T2^ labels HSC populations in healthy and fibrotic liver

**Figure 2:**
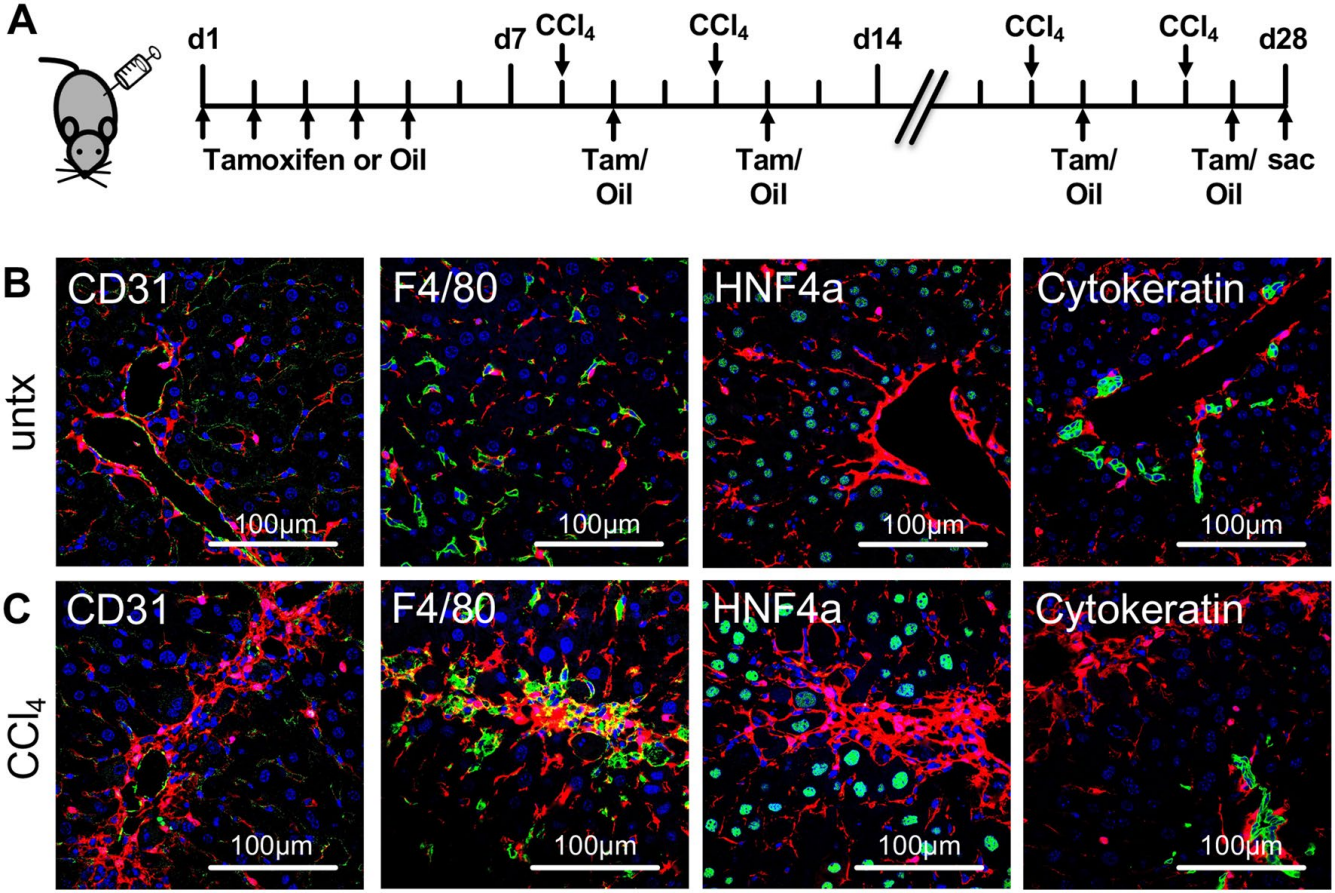
Tamoxifen induced tdTomato expression in PDGFRβ-P2A-CreER^T2^ mice is restricted to mesenchymal cell populations. (**A**) PDGFRβ-P2A-CreERT2 was induced by tamoxifen injection (i.p.) at 75 µg/g BW for five consecutive days. Subsequently, liver fibrosis was induced by biweekly injections of CCl_4_ for a total of three weeks. Tamoxifen was continued during liver fibrosis induction. (**B,C**) Staining for markers of endothelial cells (CD31), macrophages (F4/80), hepatocytes (HNF4a), and cytokeratin show that PDGFRβ-P2A-CreER^T2^ driven tdTomato expression is exclusive to mesenchymal cell populations both in untreated and fibrotic mice.

To confirm that PDGFRβ-P2A-CreER^T2^ mediated reporter gene expression is mainly restricted to mesenchymal cell populations in the liver, frozen liver sections of PDGFRβ-P2A-CreER^T2^ x tdTomato mice were stained for markers of endothelial cells (CD31), macrophages (F4/80), hepatocytes (HNF4a), and cholangiocytes (cytokeratin) after tamoxifen induction. No overlap of Cre-induced tdTomato expression with macrophage, hepatocyte and cholangiocyte markers was observed (Fig. 2A,B). Furthermore, staining for the aforementioned markers was performed in PDGFRβ-P2A-CreER^T2^ x tdTomato mice treated with six injections of CCl_4_, in order to analyze whether PDGFRβ-P2A-CreER^T2^ mediated reporter gene expression is restricted to mesenchymal cell populations in the injured liver as well. Similarly to untreated animals, no overlap of Cre-induced tdTomato fluorescence was observed with CD31, F4/80, HNF4a or cytokeratin positive cells (Fig. 2C), indicating that PDGFRβ-P2A-CreER^T2^ does not label endothelial cells, macrophages, hepatocytes or cholangiocytes in the fibrotic liver.

### PDGFRβ-P2A-CreER^T2^-labelled mesenchymal cells give rise to myofibroblasts in toxic and cholestatic liver fibrosis

**Figure 3:**
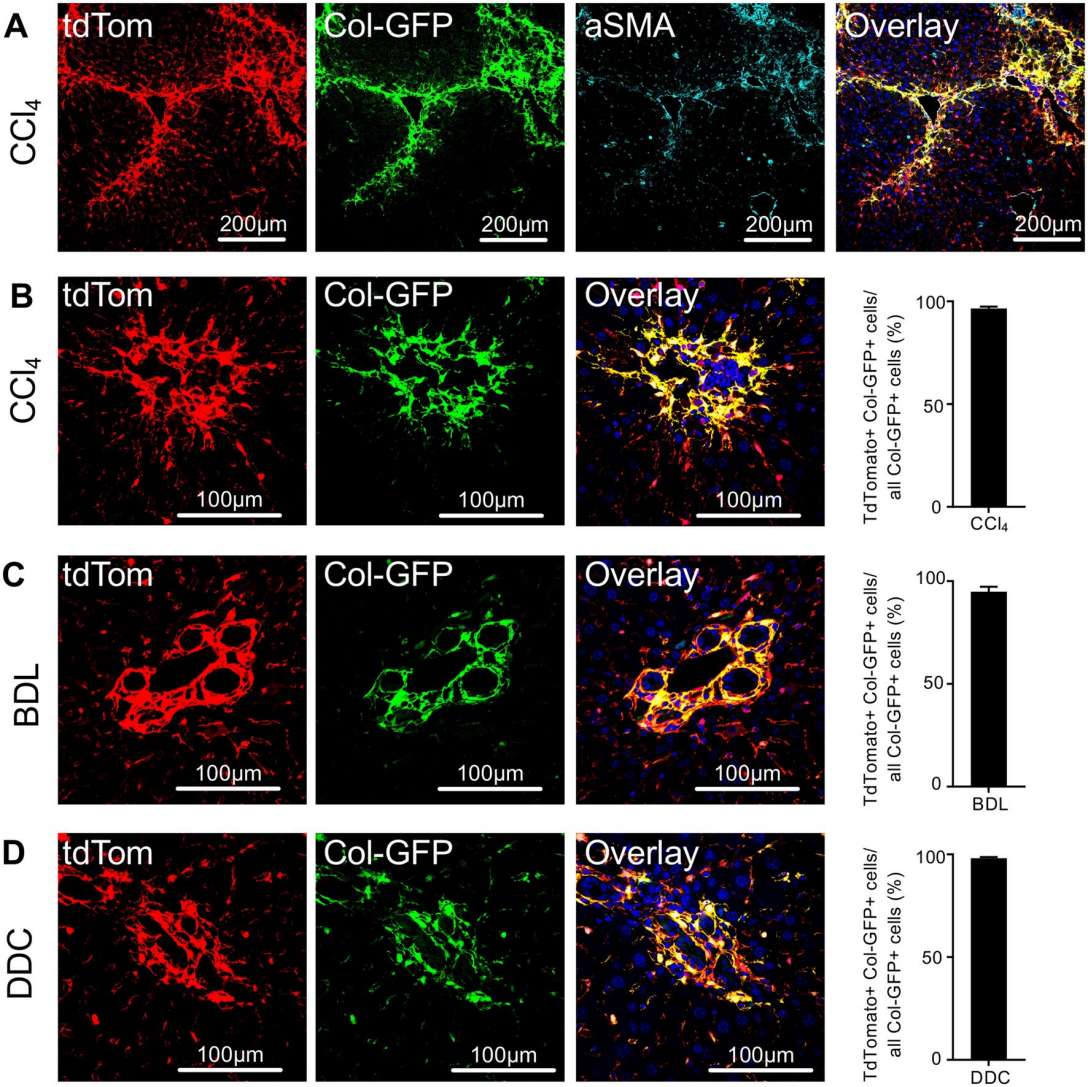
PDGFRβ-P2A-CreER^T2^ labelled mesenchymal cell populations give rise to myofibroblasts in different models of liver injury. (**A**) Staining for myofibroblast marker aSMA combined with Col1a1GFP (ColGFP) overlaps with PDGFRβ-P2A-CreER^T2^ driven tdTomato expression in fibrotic liver. (**B–D**) Co-expression of ColGFP and PDGFRβ-P2A-CreER^T2^ driven tdTomato in different liver fibrosis models (CCl_4_ (n = 4), bile duct ligation BDL (n = 4), 3,5-diethoxycarbonyl-1,4-dihydrocollidine DDC (n = 4)). Means ± SEM.

Next, we were interested whether PDGFRβ-P2A-CreER^T2^ x tdTomato labelled mesenchymal cells in the liver transdifferentiate into myofibroblasts. Therefore, PDGFRβ-P2A-CreER^T2^ x tdTomato were crossed with mice expressing collagen 1a1 driven GFP (ColGFP). In these triple transgenic mice, we observed a strong overlap of PDGFRβ-P2A-CreER^T2^ driven tdTomato expression with ColGFP (marking myofibroblasts)^12^ and almost complete overlap of those cells with the fibroblast marker alpha smooth muscle actin (aSMA) (Fig. 3A). This data confirms, that the PDGFRβ-P2A-CreER^T2^ labelled cells give rise to the majority of Col1a1 positive fibrogenic cells in the liver.

To quantify the amount of PDGFRβ-P2A-CreER^T2^-derived myofibroblasts, we used the triple transgenic PDGFRβ-P2A-CreER^T2^ x tdTomato x Col1a1-GFP mouse and induced liver fibrosis by CCl_4_. Using this approach, we observed that over 95% of Col1a1-positive myofibroblasts also expressed PDGFRβ-P2A-CreERT2 induced tdTomato indicating that almost all myofibroblasts in this model were derived from PDGFRβ-expressing mesenchymal cell populations (Fig. 3B). To exclude that the data may be specific to toxic liver fibrosis, two well-established models of cholestasis-induced liver fibrosis, bile duct ligation and 3,5-diethoxycarbonyl-1,4-dihydrocollidine (DDC)-containing diet, were employed. Similar to CCl_4_, we observed a strong overlap of PDGFRβ-P2A-CreERT2 induced tdTomato expression and Col1a1-GFP indicating that the observed effects are independent of etiology of liver damage (Fig. 3C,D). Additionally, staining for Thy1.2^13^ and Slit2^14^ was performed as markers of portal fibroblasts and portal fibroblasts with mesenchymal stem cell features, respectively. Thy1.2 predominantly marked cells in the portal area (Supplemental Fig. 2A–C). In the CCl_4_ fibrosis model, approximately half of the Thy 1.2 positive cells were also labelled by PDGFRβ-P2A-CreER (Supplemental Fig. 2B). In liver fibrosis induced by bile duct ligation, the majority of Thy 1.2 positive cells in the periportal area were also labelled by PDGFRβ-P2A-CreER (Supplemental Fig. 2C). Slit2 was expressed on PDGFRβ-P2A-CreER^T2^ positive cells, both in the liver parenchyma as well as in the portal areas (Supplemental Fig. 3A–C).

To exclude, that the continuation of Tamoxifen injections during liver injury overestimated Cre efficiency, we also analyzed overlap of PDGFRβ-P2A-CreER^T2^ induced tdTomato expression and Col1a1-GFP in animals that received the last Tamoxifen injection prior to the induction of liver fibrosis by CCl_4_. In these animals, we observed that 93.5% of Col1a1-positive myofibroblasts also expressed PDGFRβ-P2A-CreER^T2^ induced tdTomato indicating a similar degree of PDGFRβ-P2A-CreER^T2^ labelled cells in animals with or without continuation of Tamoxifen during liver injury (Supplemental Fig. 4).

## Discussion

The role of mesenchymal cell populations in the development of liver fibrosis has been extensively studied in the last decades^15–19^, but oftentimes interpretation of the results is limited by the models used.

Both the Lrat- and the PDGFRβ promoters were shown to label HSCs in the context of liver fibrosis of different etiologies^3,7^. However, both the previously described LratCre and PDGFRβ-Cre reporter mice have a constitutively active Cre recombinase, which limits their use. This can result in unspecific activity if the promoter is only temporarily active in the course of cell differentiation, but not specific for the differentiated cell^20^. Furthermore, the time point of Cre activity can be a concern once a specific deletion results in cell lethality during early development.

Those concerns could be addressed by using a transgenic mouse model with inducible Cre expression. However, no such model that targets mesenchymal cell populations has been published so far. We therefore investigated whether the inducible PDGFRβ-P2A-CreER^T2^ mouse^11^ can be used as a reliable tool to express transgens in mesenchymal cell populations in the liver.

To address this question, we generated a triple transgenic mouse model containing the PDGFRβ-P2A-CreER^T2^^11^, a red fluorescent tdTomato Cre reporter^21^, and a Col1a1-driven GFP^22^. Our data revealed that tamoxifen-induced activation of PDGFRβ-P2A-CreER^T2^ efficiently induced reporter gene expression in pericytes of the liver, which lasted up to one year after activation. Experiments with vehicle-treated mice showed practically no reporter expression, demonstrating no significant leakiness of the PDGFRβ-P2A-CreER^T2^ construct which has been observed for other CreER transgenic mice, such as the RipCreER, a beta-cell specific mouse line that can be used to manipulate gene expression in insulin-producing cells of the endocrine pancreas^23^. PDGFRβ-P2A-CreER^T2^ induced reporter expression was tested via immunostaining for markers of different liver resident cell types, in which fluorescent reporter expression only overlapped with desmin, a marker commonly used to stain pericytes in different organs, including HSCs^3,24–26^. Staining for the portal fibroblast marker Thy1.2 revealed overlap with PDGFRβ-P2A-CreER^T2^ induced reporter expression, which is in line with previous data showing that fibroblasts and VSMC express Thy1 to a certain extent^5^. It has been suggested that HSCs and Thy1.2 positive cells are two distinct cell populations^13,27^, however it cannot be excluded that also some HSCs express Thy1^5^.

Furthermore, PDGFRβ-P2A-CreER^T2^ induced reporter expression remained specific for mesenchymal cells even under fibrogenic conditions in the CCl_4_ toxic liver fibrosis model. Overlap of alpha smooth muscle actin, a common myofibroblast marker, and overlap with endogenous collagen 1a1 driven GFP further confirmed that fibrogenic cells in the liver are PDGFRβ-P2A-CreER^T2^ derived. As we achieve a high recombination of PDGFRβ-P2A-CreER^T2^ in retinoid positive HSCs of over 90% and a similarly high percentage of pericyte derived myofibroblasts in three different liver fibrosis models, the inducible PDGFRβ-P2A-CreER^T2^ model can be used once an inducible Cre mouse model for liver mesenchymal cell populations is required with a similar efficiency as the constitutive PDGFRβCre^7^ or the well accepted LratCre transgenic mouse model^3^.

However, PDGFRβCre as a marker for fibrogenic cells in the liver has some limitations. In recent years, single cell RNA sequencing studies using a Pdgfrb-GFP transgenic mouse have revealed a spatial zonation of HSCs with central-vein-associated HSCs and portal vein-associated HSCs, whereby central-vein-associated HSCs were the dominant collagen-producing cells in CCl_4_ induced toxic liver injury^5^. Without other markers, PDGFRβCre cannot distinguish between these different HSC populations with distinct functions and the different PDGFRβ-positive cell populations including fibroblasts, HSCs and VSMC^5^. Furthermore, another study identified several clusters of fibroblasts in the liver, with some of them (Fib-3 and Fib-4 clusters) expressing low levels of Pdgfrb and thus being underestimated in studies using Pdgfrb as a promoter for Cre recombinases or GFP^14^. We also performed immunohistochemistry for Slit2, a marker for portal fibroblasts with mesenchymal stem cell features (PMSCs)^14^. In contrast to this study, we observed Slit2 expression not only restricted to the portal area, but also in the liver parenchmya. Of note, PDGFRβ-P2A-CreER^T2^ driven tdTomato expression overlapped with Slit2, both in normal and fibrotic liver indicating that Slit2 might not only be a precursor for PMSCs but also for HSCs. Further studies need to address this finding.

Nevertheless, our data demonstrates that the tamoxifen inducible PDGFRβ-P2A-CreER^T2^ mouse model is similarly efficient to the established constitutive LratCre and PDGFRβ-Cre mouse models and can be applied once an inducible Cre recombinase is required to study liver fibrogenesis.

## Material and methods

### Mice

All animal experiments were conducted under the approval of the Lower Saxony State Office for Consumer Protection and Food Safety (LAVES, Germany; #18/3059), and in compliance with both the regulations of the German Animal Welfare Act, as well as the ARRIVE (Animal Research: Reporting of In Vivo Experiments) guidelines and regulations^28^.

PDGFRβ-P2A-CreERT2 mice (Jax #030201)^11^, and tdTomato Cre reporter mice (Jax #007909)^21^ were purchased from Jackson Laboratory. The Col1a1-GFP mouse line has been previously described^12^. For breeding, we used PDGFRβ-P2A-CreERT2 Cre-positive males and Cre negative females. Cre activity was induced by tamoxifen injection (75 µg/g BW intraperitoneal (i.p.), dissolved in maizeoil (Carl Roth) for five consecutive days (Fig. 1A). Oil injected animals served as controls. In experiments where liver injury was induced, the injection of tamoxifen was continued two times a week until the end of the respective experiment.

### Liver fibrosis and injury models

Toxic liver fibrosis was induced by biweekly injections of CCl_4_ (0.5 µl/g BW, dissolved in maizeoil at a ratio of 1:3) for three weeks (6 injections total). For the induction of cholestatic liver fibrosis, mice underwent ligation of the common bile duct^29^. Briefly, after abdominal incision, the common bile duct was ligated distally. As an additional model of cholestatic liver fibrosis, mice were fed a 0.1% DDC-containing diet for 3 weeks^3^. At the end of the experiments, the animals were sacrificed by an overdose of a mixture of ketamine and xylazine (300 µg ketamine + 12 µg xylazine per g BW, i.p.) followed by cervical dislocation, after which organs were harvested for further analysis.

### Isolation of hepatic stellate cells and FACS analysis

HSCs were isolated as previously described^30^. Subsequently, freshly isolated, gradient-purified HSCs were subjected to FACS analysis. HSCs contain vitamin A with a specific fluorescence that can be detected using ultra-violet light (405–407-nm laser) for excitation and a 450/50-nm band-pass filter for detection^30^. TdTomato was analyzed using an excitation wavelength of 560-nm and was detected at 610-nm. The gating strategy is shown in Supplemental Fig. 1A. FACS analysis was performed using a MoFlo XDP Cell Sorter (Beckman-Coulter).

### Immunohistochemical staining and microscopy

Tissue from mouse liver was collected, fixed with 4% paraformaldehyde, embedded and frozen in Tissue-Tek^®^ O.C.T.™ Compound and cut to yield 7 µm sections. Frozen liver sections were incubated with the following primary antibodies: desmin (1:200, R&D, AF3844), CD31 (1:100, Abcam, ab28364), F4/80 (1:100, Thermo, BM8), HNF4a (1:100, Cell Signaling, #3113S), cytokeratin (1:200, TROMA-III), aSMA (1:100, Abcam, ab5694), Thy1.2 (1:100, Thermo, 53-2.1) and Slit2 (1:100, Abcam, ab7665). Fluorescent secondary antibodies with different fluorescent conjugates (goat anti-rat 488 (Invitrogen, A11006), 1:200; goat anti-rabbit 488 (Invitrogen, A11034), 1:200; donkey anti-rabbit Cy5 (Novus Biologicals, NBP1-75286PECY55), 1:100; donkey anti-goat 488 (Invitrogen, A11055), 1:200) were employed followed by Hoechst 33258 staining (Sigma, B2883-100MG). All immunohistochemistry- and immunofluorescence-based quantification was performed on sections containing representative tissue from several lobes of the liver (three midsized tissue pieces per liver per mouse). Fluorescence images were captured employing a Nikon eclipse Ti2 microscope or DMi8 confocal laser microscope (Leica). Images were analyzed using ImageJ software (Version 1.51n).

## Supplementary Information

Supplementary Information.

## Data Availability

All data generated or analyzed during this study are included in this published article (and its Supplementary Information files).
