# Supplementary figures and images for: An inducible model for genetic manipulation and fate-tracing of PDGFRβ-expressing fibrogenic cells in the liver
Source: Sci Rep. 2023 May 5;13:7322. doi: 10.1038/s41598-023-34353-y (PMC10162963; doi:10.1038/s41598-023-34353-y)

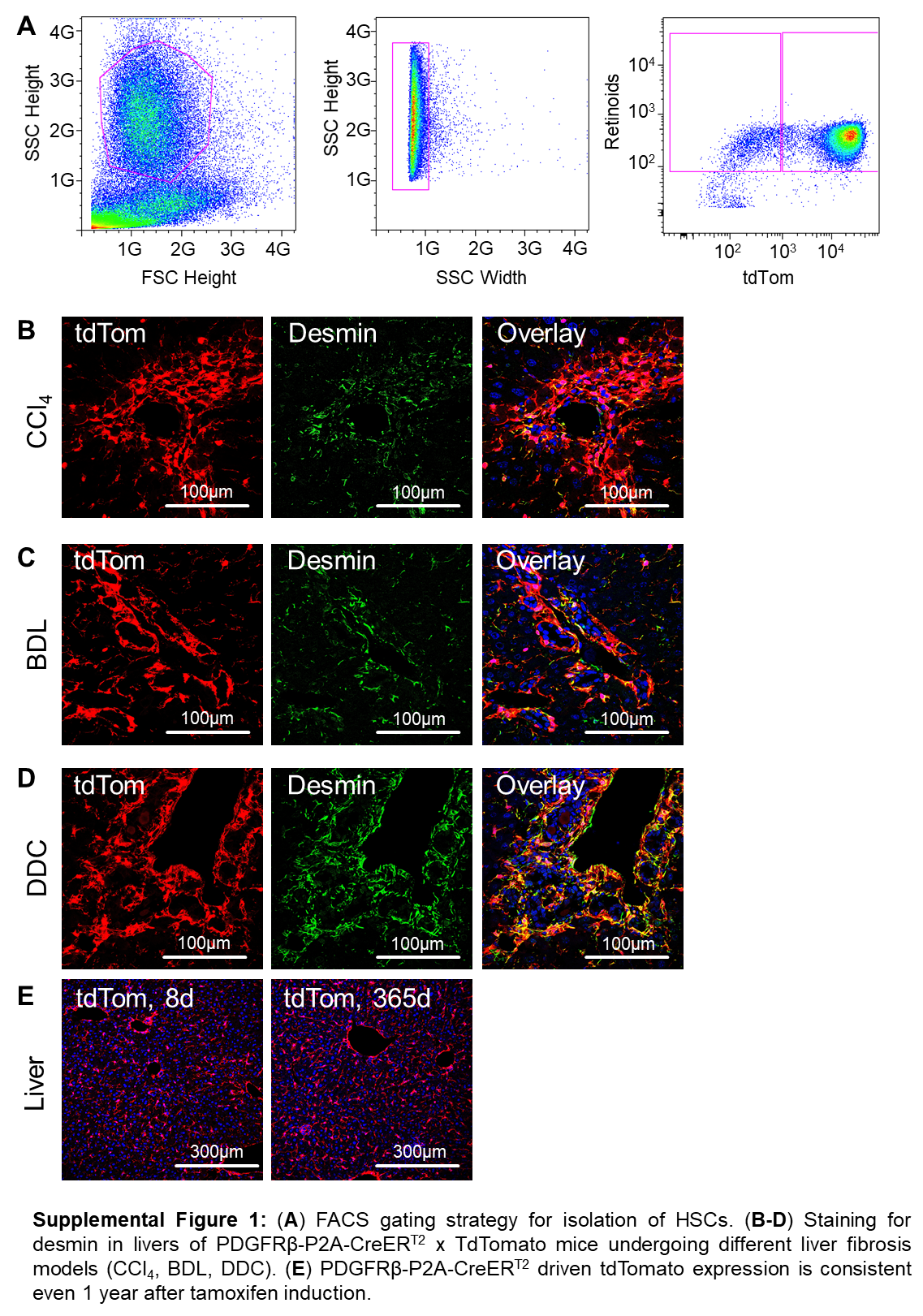


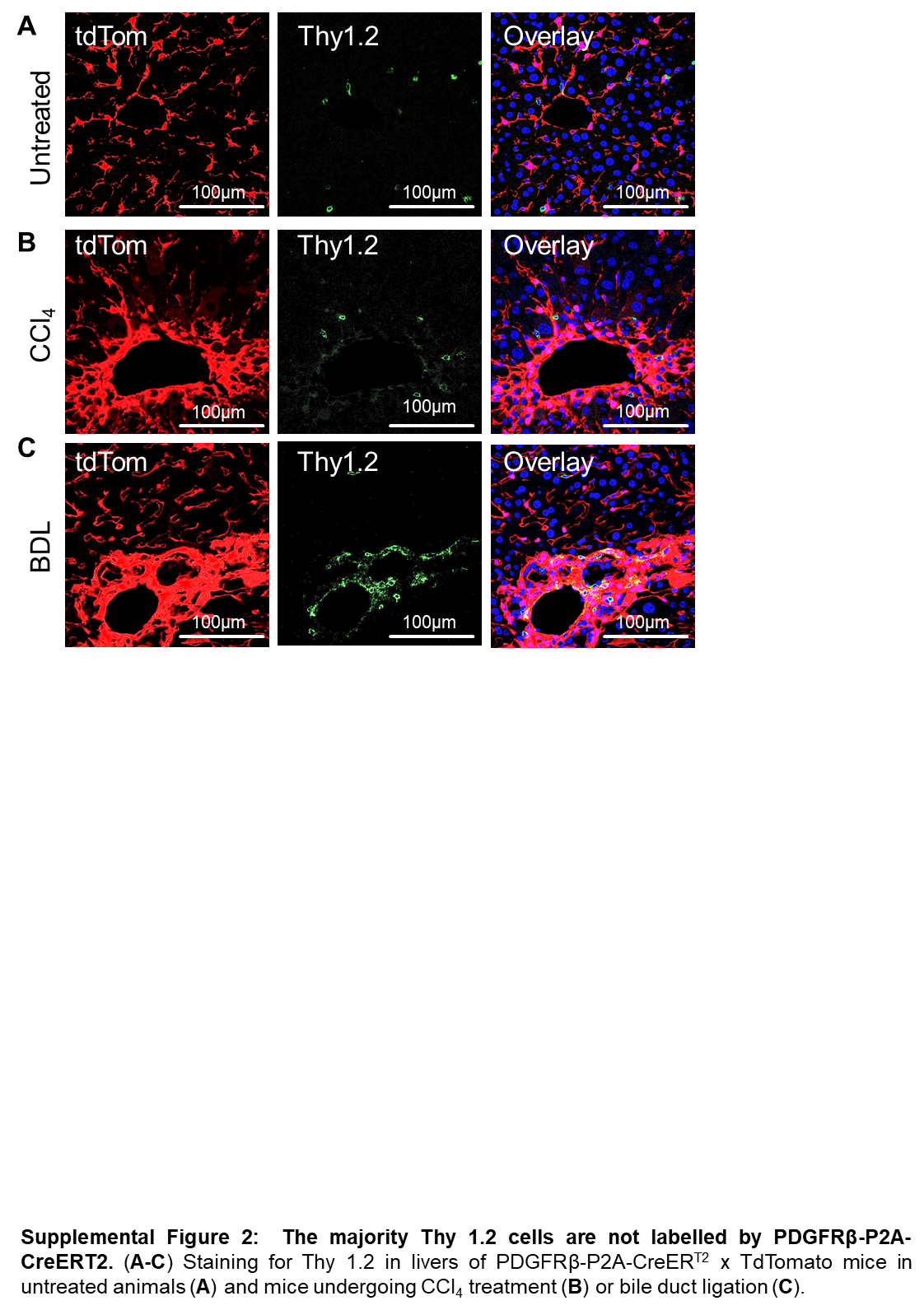


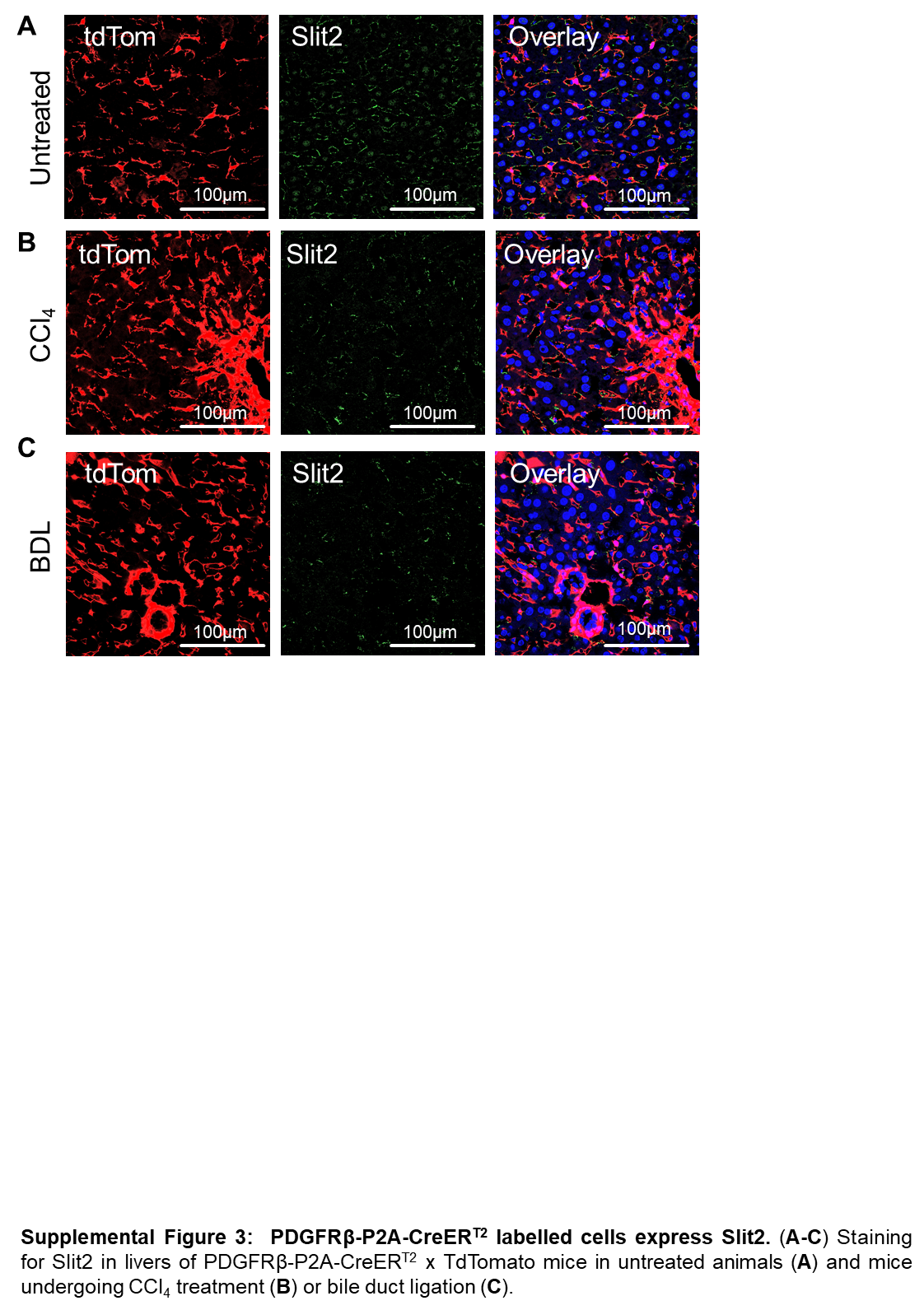


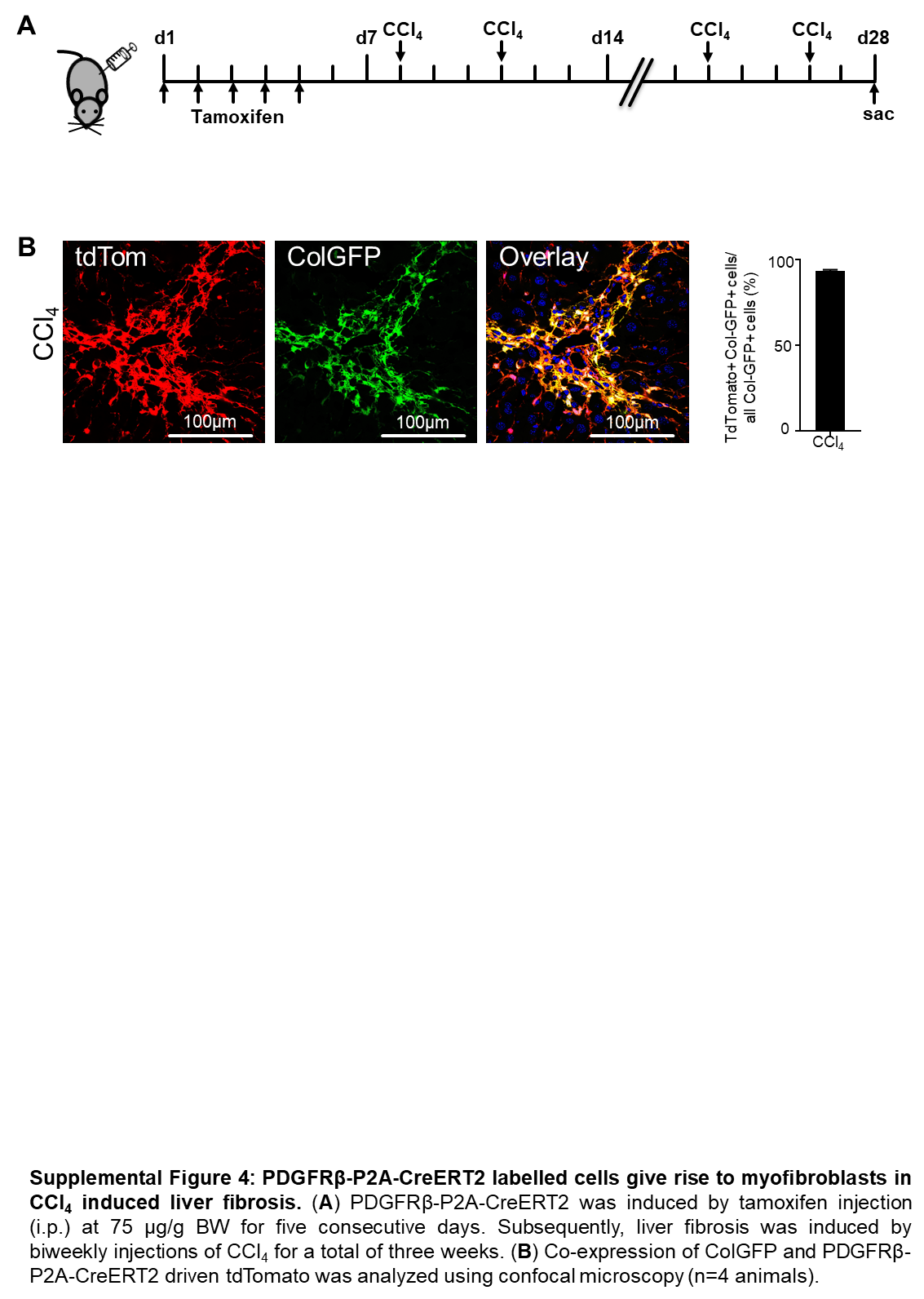

Supplement: Supplementary file 1 — Supplementary Information. [file 41598_2023_34353_MOESM1_ESM.docx]
